# Bearing-Fault Diagnosis with Signal-to-RGB Image Mapping and Multichannel Multiscale Convolutional Neural Network

**DOI:** 10.3390/e24111569

**Published:** 2022-10-31

**Authors:** Ming Xu, Jinfeng Gao, Zhong Zhang, Heshan Wang

**Affiliations:** College of Electrical Engineering, Zhengzhou University, Zhengzhou 450001, China

**Keywords:** fault diagnosis, multichannel and multiscale convolutional neural network (MCMS-CNN), rotating machinery, sensor fusion, signal-to-RGB image mapping (STRIM)

## Abstract

Deep learning bearing-fault diagnosis has shown strong vitality in recent years. In industrial practice, the running state of bearings is monitored by collecting data from multiple sensors, for instance, the drive end, the fan end, and the base. Given the complexity of the operating conditions and the limited number of bearing-fault samples, obtaining complementary fault features using the traditional fault-diagnosis method, which uses statistical characteristic in time or frequency, is difficult and relies heavily on prior knowledge. In addition, intelligent bearing-fault diagnosis based on a convolutional neural network (CNN) has several deficiencies, such as single-scale fixed convolutional kernels, excessive dependence on experts’ experience, and a limited capacity for learning a small training dataset. Considering these drawbacks, a novel intelligent bearing-fault-diagnosis method based on signal-to-RGB image mapping (STRIM) and multichannel multiscale CNN (MCMS-CNN) is proposed. First, the signals from three different sensors are converted into RGB images by the STRIM method to achieve feature fusion. To extract RGB image features effectively, the proposed MCMS-CNN is established, which can automatically learn complementary and abundant features at different scales. By increasing the width and decreasing the depth of the network, the overfitting caused by the complex network for a small dataset is eliminated, and the fault classification capability is guaranteed simultaneously. The performance of the method is verified through the Case Western Reserve University’s (CWRU) bearing dataset. Compared with different DL approaches, the proposed approach can effectively realize fault diagnosis and substantially outperform other methods.

## 1. Introduction

As one of the most critical components of a rotary mechanical system, the malfunction of rolling bearings seriously influences the reliability and the stability of equipment or even threatens equipment running time and human safety [1]. In actual industry, the heat-generation rate of cylindrical roller bearings, the damage of rolling bearings, etc., can greatly affect the operation status of industrial systems [2,3]. Therefore, the accurate and feasible diagnosis of bearing faults is essential in modern industry [4,5]. Various bearing-fault-diagnosis methods have been studied in the academic and engineering fields. In general, the methods can be simply divided into model-based and data-driven approaches [6]. Model-based methods strongly depend on physical process knowledge. Specifically, a complex industrial system usually has the comprehensive characteristics of strong coupling, time delay, and multiple parameters. Establishing an accurate analytical models when the system is complicated is often impossible. For modern complex mechanical equipment, considerable nonlinear and high-dimensional data can be collected from many kinds of sensors. Data-driven methods only depend on the historical data for the process and extract the useful features from the state of the process. These extracted features can be divided into time domain, frequency domain and time-frequency domain features. Time domain features mainly focus on the analysis of the statistical values of a vibration signal. In ref. [7], the sensitive statistical values for different bearing-fault locations are clearly discussed. Frequency domain features mainly consider the frequency domain components of the signal, such as its spectrum. Wavelet transform and empirical mode decomposition are usually adopted to obtain time-frequency features. Therefore, data-driven fault-diagnosis methods are becoming appropriate even for complicated systems.

Machine learning (ML), as a representative data-driven fault-diagnosis method, has a strong capability of fault information extraction and fault classification [8]. Among ML methods, many techniques for signal processing and information extraction in fault conditions have been reported and discussed in the past few years. Features, i.e., signals in time, frequency, and the time-frequency domain, have been studied and utilized for bearing-fault diagnosis [9]. He et al. [10] introduced a time domain signal-processing method that embedded vibration-analysis techniques into a deep learning (DL) structure for carrying out fault classification. Muhammad et al. [11] employed a novel vibration spectrum imaging (VSI) method to translate vibration signals into spectral images. They then utilized an artificial neural network (ANN) as a fault categorizer to realize defect diagnosis. Wang et al. [12] converted 1D time sequences of different gearbox fault conditions into time-frequency images. In addition, a novel approach named deep convolutional neural network (DCNN) was investigated for fault identification under various working conditions. Zhang et al. [13] incorporated signals in time and the frequency domain through short-time Fourier transform (STFT) to obtain time-frequency images. These images were used as inputs to a CNN to determine the fault types. A novel time-frequency spectrum image-conversion method based on the Hilbert–Huang transform (HHT) was proposed. It was combined with a probabilistic neural network (PNN) to diagnose bearing-fault conditions efficiently [14].

The abovementioned signal-processing methods manually extract several time domain, frequency domain, or time-frequency domain features of raw vibration signals for bearing-fault diagnosis [15]. Although these methods have achieved meritorious results, they usually rely on much diagnostic expertise and knowledge for feature-frequency calculation and have a poor ability to be generalized [16]. Regardless of the kinds of signal-to-image transformation methods, information attenuation occurs. Many features obtained through transformation are only valid for specific conditions and stages. To overcome these problems, a signal-to-RGB image mapping (STRIM) method, that is, the fusion of raw data from multiple sensors to generate RGB images, is proposed. This method can directly utilize the original signal and retain the full information of the signals of all sensors. Through this method, each type of fault is presented in the form of RGB images. Each pixel of the RGB image contains the data of all the sensors associated with the fault condition, enabling the full display of the fault conditions.

With the rapid development of ML, DL is increasingly applied to overcome the insufficient feature-extraction capability of signal-processing methods [17]. Many DL methods [18,19], such as deep belief network (DBN), sparse autoencoder (SAE), stacked denoising autoencoder (SDAE), sparse filtering, and CNN have been presented and applied in fault classification. Xing et al. [20] designed a distribution-invariant DBN for fault recognition, which can directly extract features of a raw vibration signal. She et al. [21] presented an improved-regularization SAE model for rolling bearings. Chen et al. [22] applied SAE to fuse time domain and frequency domain sensor signals. Then DBN was proposed for identifying bearing fault. Chen et al. [23] applied a CNN based on an auto-encoder to implement feature selection from unlabeled data automatically. The results showed its strong robustness and excellent adaptability. Enhanced sparse filtering was investigated to eliminate the effect of environmental noise [24]. Fast convolutional sparse filtering based on convolutional activation and feature normalization was proposed for gearbox fault diagnosis [25]. Among these DL methods, the architecture based on CNN has achieved outstanding performance [26]. In ref. [27], intelligent fault diagnosis through combining STFT and CNN was introduced. Zou et al. [28] employed an improved adversarial denoising CNN to overcome the limitation of fault data and the influence of noise in real industry. To overcome the imbalanced distribution of mechanical conditions, a deep-normalized CNN was utilized to classify fault types [29]. Hoang et al. [30] carried out a method for identifying fault types based on CNN using vibration signals directly.

Although the above DL methods have shown powerful functions in fault identification and classification, these methods often require various data for training, which necessitates a deep or complex network [31]. In a practical industrial process, a large amount of data under normal operating conditions is collected, and the collection of fault data is often limited [32]. Consequently, fewer data render the model unable to be trained well or even give it a tendency to overfit, which leads to poor classification results [33,34]. Moreover, the abovementioned CNN-based diagnosis methods do not take the convolutional kernel size within different convolutional channels into account. Fixed-size convolution kernels are unsuitable for complex working conditions and changing loads because the signals are usually obtained from different sensors at different sampling frequencies [35]. Therefore, a fault-diagnosis model that fully considers the signal under complex working conditions can have a better generalization ability in an industrial system [36].

To eliminate these influences, a new intelligent bearing-fault-diagnosis model is designed in this article. First, a novel STRIM method is introduced to achieve sensor data fusion by converting the raw vibration signal of multiple sensors into RGB images without any predefined parameters. An MCMS-CNN is then introduced to realize bearing-fault diagnosis. A multichannel and multiscale strategy is designed to extract multiscale fault-feature information. Three convolution kernels of different scales and convolution channels are selected, and the features of the same input vibration signals are obtained from different scales in parallel. Convolution kernels of different sizes have distinct receptive fields, resulting in a comprehensive feature space contained in the convolution of various scales of kernels. In different categories of fault images, the more information obtained, the higher the accuracy and the stability of the prediction will be. Therefore, convolution kernels of different scales should be added in the convolution layer.

The main contributions of this paper can be summarized as follows:

1. A new STRIM method is introduced to convert a raw signal into RGB images without any predefined parameters. This conversion method overcomes the difficulty of extracting features by manual feature learning and the need for numerous experts’ experiences. This method cannot only utilize the original signal directly but can also integrate the data of all the sensors and maximally retain full information of all the sensors’ signals.
2. A novel intelligent fault-diagnosis method under different failure states is proposed based on MCMS-CNN. The multichannel and multiscale strategy can learn complementary and abundant feature information at diverse scales and reduce the depth of the network.
3. Compared with signal-scale-based CNN methods, the proposed MCMS-CNN method is more generalized and efficient.

The remaining part of this article is organized as follow: Section 2 introduces the theoretical background and limitation. Section 3 describes the proposed method in detail, whereas Section 4 introduces and analyzes the experimental results, along with a discussion of the proposed method. A brief conclusion is summarized in Section 5

## 2. Theoretical Background

The theoretical background is described in this section, which contains the STIM, CNN, and dropout. Some limitations are also discussed for STIM and CNN.

### 2.1. Signal-to-Image Method (STIM)

**Figure 1:**
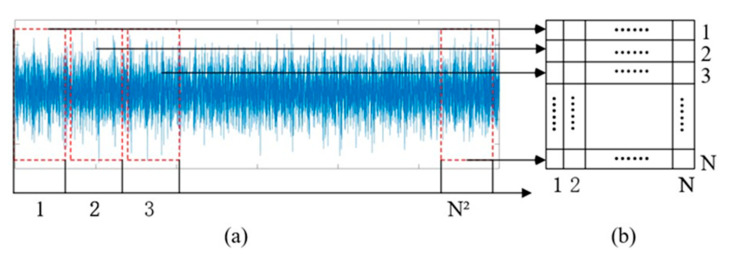
Signal-to-image: (**a**) Time-domain signal; (**b**) Grey image.

In STIM [37], the raw 1D signal is translated into 2D gray image directly as shown in Figure 1. Gray images are obtained by filling the pixels through the original signal sequentially. Once the length $N^{2}$ of the data is determined, the N×N size image is acquired. Let $L\left(i\right),\;i=1,\cdots ,\;N^{2}$ be a set of different data. P(j,k),j=1,⋯N,k=1,⋯N. P(j,k) is the pixel value of gray pictures. The specific process is expressed as follows:

(1) (j,k)=L((j−1)×N+k)×255)

The pixel value represents the value of the raw signal. Each value of the original data is normalized from 0 to 255 by Equation (1), and the size of the grey image relies on the volume of signal data.

Analysis shows that the STIM does not require any expert experience, prior knowledge, or complex preprocessing methods. This method directly uses the 1D raw signal to form 2D gray images. Therefore, this method provides an idea of visualizing the bearing vibration signal.

The limitations of the STIM include the following:

1. The original signal is tiled row by row into a matrix to form a gray image. Each pixel of the gray image contains only one fault-related sensor data, which cannot adapt to the fault conditions associated with various sensors.
2. From the image presentation, the STIM only uses gray images to represent fault data, which is relatively simple and contains incomplete fault information. To use the gray images generated by the STIM for fault diagnosis, a powerful feature-extraction and classification algorithm is required.

### 2.2. CNN

In this section, the fundamental theory and the limitations of CNN are introduced.

#### 2.2.1. Convolutional Layer (C-Layer)

In C-layers, feature maps are generated by convolving an input image with different sizes of convolutional kernel. Given that the feature maps have similar statistical characteristics, the required features can be rearranged and mined through a series of convolutions. After the convolution, feature extraction is obtained by an activation function [38]. The operation of convolution is defined in Equation (2):

(2) $$X_{j}^{l}=\varphi (\sum_{k}x_{k}^{l-1}*\omega_{kj}^{l}+b_{j}^{l})$$

where ∗ means convolution operator. $X_{j}^{l}$ represents the jth feature map of layer l; $x_{k}^{l-1}$ denotes the kth output feature map of former layer. $\omega_{kj}^{l}$ and $b_{j}^{l}$ are the convolutional kernel and bias. φ is a nonlinear activation function.

#### 2.2.2. Pooling Layer (P-Layer)

Following the C-layer is the pooling layer. The purpose of the pooling layer is to decrease redundant features by downsampling operation. Through downsampling, the parameters of the network are reduced, and the calculation efficiency is improved. Max-pooling is utilized, which is given as follows [38]:

(3) $$X_{j}^{l}=f\left(\beta_{j}^{l}\;*\;down\left(x_{j}^{l-1}\right)+b_{j}^{l}\right)$$

where $\beta_{j}^{l}$ and $b_{j}^{l}$ denote the weight and bias of jth feature map of layer l. down(∗) is the downsampling function.

#### 2.2.3. Fully Connected Layer (FC-Layer)

The fully connected layer is generally added after several rounds of C-layers and P-layers. Its role is equivalent to a classifier. The extracted feature collected by the C-layers and P-layers are sequentially expanded in a feature vector and used as the input of the FC-layer. The output of the FC-layer is mostly obtained with the softmax classifier that is suitable for multi-type classification. The function of softmax is described as follows [39]:

(4) $$F_{i}=\frac{\exp\left(P_{FC}x_{i}\right)}{\sum_{j=1}^{k}\exp\left(P_{FC}x_{j}\right)}\;1\;\leq \;i\;\leq \;k$$

where $P_{FC}$ represents the parameters of the fully connected layer, $x_{i}$ is the ith input of fully connected layer, $F_{i}$ is a vector of length with range (0, 1), and k represents the k-category classification.

CNN has powerful functions in classification and feature extraction. However, CNN has the following limitation:

1. In traditional CNNs, the kernel sizes of the convolutional layers are fixed with a single scale. Consequently, the single scale module fails to learn features at different scales that do not adapt diverse fault conditions.
2. Traditional CNN often requires various data for training. For a small dataset, the traditional CNN can easily fall into the state of overfitting and cannot meet the classification requirements.
3. The improvement of the accuracy of traditional CNN often comes at the cost of increasing the depth of the network and the complexity of the network structure.

### 2.3. Drop Out

**Figure 2:**
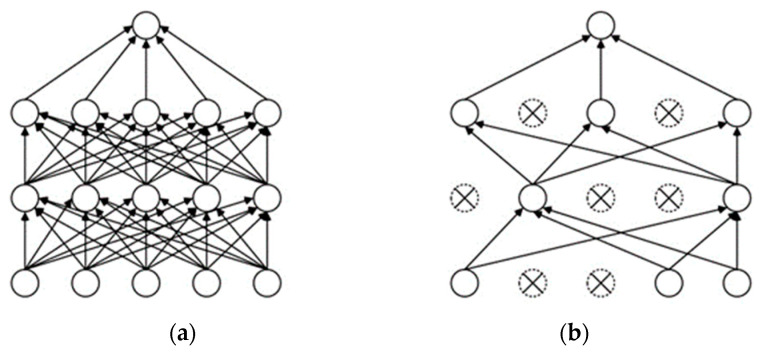
Dropout: (**a**) Standard neural network; (**b**) After applying dropout.

From the former analysis, a dropout layer is adopted after the fully connected layer to overcome the overfitting caused by limited data. The procedure of dropout is demonstrated in Figure 2. Figure 2a shows the standard neural network, and Figure 2b presents the neural network after applying dropout. During forward propagation, several neurons are randomly hidden with a certain probability and excessive mutual adaptation between neurons is prevented.

## 3. STRIM and MCMS-CNN Based Fault Diagnosis Method

Based on the aforementioned theoretical basis for STIM and CNN and existing problems, fault diagnosis based on STRIM and MCMS-CNN is proposed in this section. First, the introduction of STRIM is presented. Second, the definition of MCMS-CNN is given.

### 3.1. STRIM

In traditional diagnosis methods, the raw signal cannot be disposed directly. Preprocessing methods, which need much diagnosis expertise, are utilized. In industrial manufacturing, if one component of the equipment fails, all sensors will record the fault data at the same time. Previously, STIM used the signal of only one sensor to generate gray images, which may cause useful information from other sensors to be neglected. In order to overcome the above shortcoming, a STRIM method that can handle raw data directly by fusing the data from all sensors and converting the vibration signals into RGB images is proposed.

In image construction, the data of the sensors are normalized from 0 to 255, which is just the pixel value of the RGB image. The specific method is as follows:

(5) $$M\left(j,k\right)=round\left\{\frac{V\left(\left(j-1\right)\times N+k\right)-Min\left(V\right)}{Max\left(V\right)-Min\left(V\right)}\times 255\right\}$$

where N denotes the size of the RGB image; the size is determined according to the signal period information. Let V(i),i=1,2,⋯,N represent the value of the signal, and M(j,k),j=1,2,⋯,N, k=1,2,⋯,N denote the reshaped matrix. round(·) is the rounding function. Each pixel value is normalized from 0 to 255.

**Figure 3:**
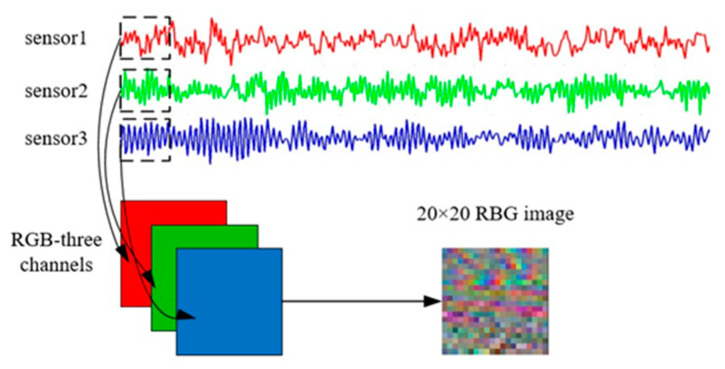
The proposed STRIM.

After the above operation is performed on the raw signals of all three sensors (the drive end [DE], the fan end [FE], and the base [BA]), the reshaped matrices of multiple sensors are placed into the R, G, and B channels, respectively, to constitute an RGB image. In the RGB images, the intensity of pixels reflects different original data values. The entire process is presented in Figure 3.

The advantages of this STRIM approach are summarized below:

1. Each channel of the RGB image corresponds to the data of a sensor, and each pixel of the RGB image contains the data of all the sensors associated with the fault condition, enabling the full display of the fault conditions.
2. The size of the RGB image is determined according to the periodic characteristics of the original data, which fuses the periodic information into the RGB image simultaneously. STRIM can use the original signal directly without any additional signal processing, maximally retaining all the fault information of the fault conditions.
3. The RGB images constructed by STRIM have a large degree of distinction between different fault conditions, laying a good foundation for feature extraction and subsequent classification.

### 3.2. MCMS-CNN

In traditional CNNs, the selection of the convolution kernel size is of great importance. However, convolution kernels of different sizes have varying receptive fields, resulting in diverse features and dissimilar information being extracted.

**Figure 4:**
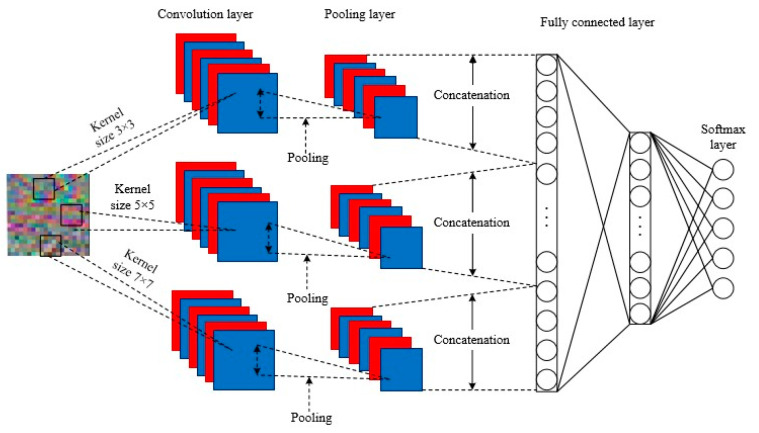
The framework of MCMS-CNN.

In this subsection, a novel MCMS-CNN is established, and the overall architecture of the MCMS-CNN is graphically illustrated in Figure 4. In the proposed MCMS-CNN, three convolution kernels of different scales are selected, and each scale of convolution kernel corresponds to a channel to perform convolution operations in parallel to extract the features of the RGB fault image at different scales. Although the three convolution calculation channels are parallel and mutually independent, they share hyperparameters during network training. To learn more abstract features from the input RGB images, the kernel sizes with different channels should cover a large range. The size of the convolution kernel should satisfy the following:

(6) $$s=2n+1\;\;\;\;n\in N^{*}$$

The kernel sizes of different channels are set to 3×3, 5×5, 7×7. The features obtained of the three parallel convolution networks are concatenated as the input of classification layers. The concatenated layer can be described as follows:

(7) $$F\left(X_{MSC}\right)=concatenate\left[Conv_{1}(X_{MSC}\right),\cdots ,Conv_{n}\left(X_{MSC}\right)]$$

where $X_{MSC}$ is the input feature, and $Conv_{i}$ represents the convolution operations of different scale convolutional kernels.

The proposed MCMS-CNN has the following two advantages:

1. Convolutional kernels of different channels have different scales, which results in the extraction of different features. Thus, the details and the global information of the same RGB image can be obtained simultaneously, enriching the features of the fault conditions. The more feature information is extracted, the more accurate the subsequent classification will be.
2. An increase in the width of the network is accompanied by a decrease in the depth of the network, and the number of convolutional layers and the complicacy of the network are reduced without affecting the classification effect.

### 3.3. Zero Padding

**Figure 5:**
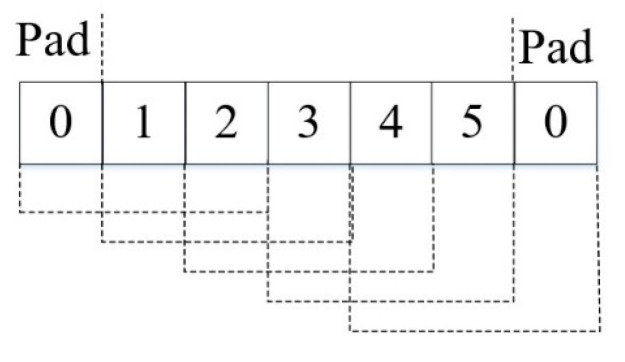
Zero padding.

To solve the inconsistency of output dimension caused by different convolution kernel sizes, zero padding [19] is utilized to overcome dimension loss, as presented in Figure 5. N and M represent the sizes of input and output, severally. F represents the width of the filter, and S is the stride. The amount of zero padding on the left (L) and right (R) can be evaluated by:

(8) $$N=ceil\left(\frac{M}{S}\right)$$

(9) PT=(N−1)×S+F−M

(10) $$L=floor\left(\frac{PT}{2}\right)$$

(11) R=PT−L

where ceil(·) and floor(·) are the ceil and floor functions, respectively.

In the zero-padding method, zeros are automatically added to the convolution process. An instance of zero padding in one dimension is shown in Figure 5. The stride of the convolutional layer is set to 1. The parameters are M=5,S=1,F=3 and the padding results are L=1,R=1,N=5. With the above method, the outputs of different scale convolution kernels are controlled at the same size.

### 3.4. Parameter Optimization

The whole parameter set of the model is optimized by the SGD algorithm. When the SGD optimization algorithm is used for model training, the model weights are only updated based on part of the training set each time to improve the training speed. Suppose the model loss function is L(W,b), for all the input data x∈X, with G groups in total; the SGD weight recursive formula is (12) [40]

(12) $$W_{j+1}=W_{j}-\alpha \sum_{g=1}^{G}\nabla_{\omega }L_{g}\left(W,b\right)$$

Among them, $W_{j}$ is the weight matrix of the jth iteration, α is the learning rate, $\nabla_{\omega }$ is the derivative operation, subscript ω means to derive the weight, and $L_{g}\left(W,b\right)$ represents the loss function of the gth group of data. The total number of groups is G.

Overfitting is a common problem in a network, especially in the case of limited training samples, which results in a poor performance on the test set [41]. This study uses the dropout method to eliminate the influence of overfitting.

**Figure 6:**
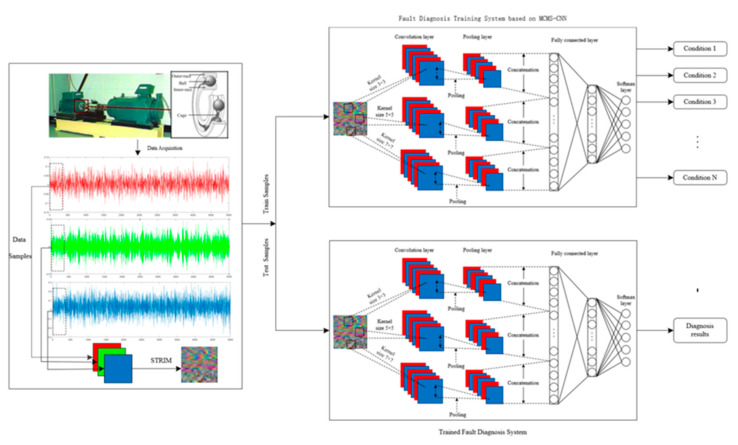
Diagnosis framework of bearings based on STRIM and MCMS-CNN.

Based on these preceding analysis, the overall architecture of the proposed bearing-fault-diagnosis system is demonstrated in Figure 6. This system includes the following five implementation steps:

Step 1: The bearing vibration signals collected from different sensors are normalized.

Step 2: The normalized signals from different sensors are converted into RGB images with a size of 20 × 20 by STRIM to realize multisensor information fusion.

Step 3: The converted images are stochastically split into train set, validation set, and test set.

Step 4: The MCMS-CNN is developed. In this process, model parameters are optimized, and feature extraction at different scales and fault classification are completed synchronously.

Step 5: The testing dataset is applied to the well-trained fault-diagnosis system for fault recognition.

## 4. Experiment and Result Analysis

In this section, the CWRU bearing dataset is examined to validate the effectiveness of the proposed STRIM and MCMS-CNN for bearing-fault classification. Through a series of comparative experiments, the superiority of the proposed method is analyzed from multiple perspectives.

### 4.1. Experimental Description

**Figure 7:**
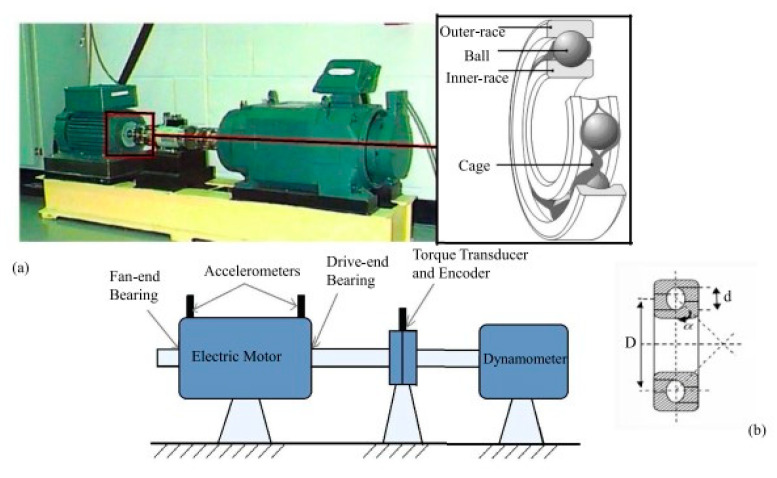
(**a**) An experimental platform of the CWRU bearing test rig for bearing system; (**b**) its cross-sectional view.

The diagnostic validity of the proposed model is carried out on the most famous public dataset, which is the CWRU bearing dataset. The experimental rig of CWRU is illustrated in Figure 7, which consists of a test motor (left), a torque transducer, and a dynamometer. The bearing vibration signals are obtained using accelerometers, which are installed on the bearing housing, mounted at three different positions, namely, DE, FE, and BA. Bearing faults are tested in this platform, including a normal condition and three defects. The defects are ball defect (BD), outer race defect (OR), and inner race defect (IR). Each of the fault types contains three fault diameters of 0.18, 0.36, and 0.54 mm. Among the OR, three locations were selected, that is, 3, 6, and 12 o’clock [42]. Therefore, 13 bearing fault conditions are obtained in this dataset, as shown in Table 1.

**Figure 8:**
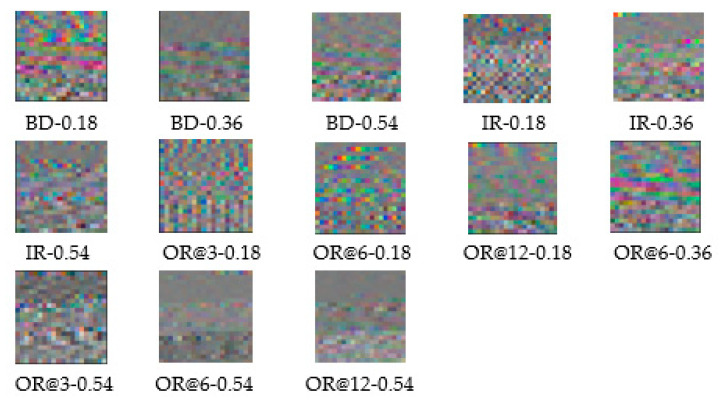
Converted RGB images on 13 fault conditions.

The original signals of the three sensors (BA, DE, and FE) are processed by STRIM. The processed sensor BA data are then fed into the R channel, the processed sensor DE data are fed into the G channel, and the processed sensor FE data are fed into the B channel. The sampling frequency of the data is 12 kHz and the rotation speed is 1797 r/min. Hence, approximately 400 ((60/1797) × 12,000 ≈ 400) points are collected per second. This method uses a 20 × 20 RGB image. Each sensor selects 120,000 samples, which can generate 300 images under one load. For the whole CWRU bearing dataset, there are four load conditions. Under each load, there are 13 fault types, so 15,600 (300 × 13 × 4) images can be obtained. The RGB images, converted using the STRIM method, of 13 types of fault conditions under 0 hp load are shown in Figure 8. Figure 8 demonstrates that these RGB images generated by STRIM are characterized by low resolution, small size, having only color pixels, and the presence of texture information, and thus are quite different from the images used by CNN for classification. The images used for traditional CNN classification are mostly images with realistic significance, which not only have high resolution and a large size, but also contain clear image features and rich semantic information. The traditional CNN model can easily fall into overfitting when dealing with the above RGB images.

After obtaining these RGB images of different fault types, the images should be divided into a training dataset and testing dataset. The training set is then fed into the proposed model for model training and the classification effect is evaluated on the testing set. At present, the common division method is to select all the fault data under the same load and divide them into a training set and test set according to the fault type. In fact, this division scheme is subject to similar biased classification results [43]. The reason for this similar bias problem is that the data used for training and testing come from the same working condition, which results in an over-optimistic classification effect. In ref. [43], the limitations and drawbacks of the above division scheme are explained in detail. Based on the above-mentioned literature, data from load 1, 2, and 3 are combined for the training set, and data from load 0 are used for the testing set, which is named dataset1. The data from loads 0, 2, and 3 for the training set and the data from load 1 for the testing set are merged and named dataset2. By that analogy, dataset3 and dataset4 are obtained. This partition ensures that the data used for training and testing are completely separated. Each dataset contains 13 types of faults. Dataset1 is taken as an example to give the specific division results, which are shown in Table 2.

### 4.2. Performance of the Proposed MCMS-CNN

**Figure 9:**
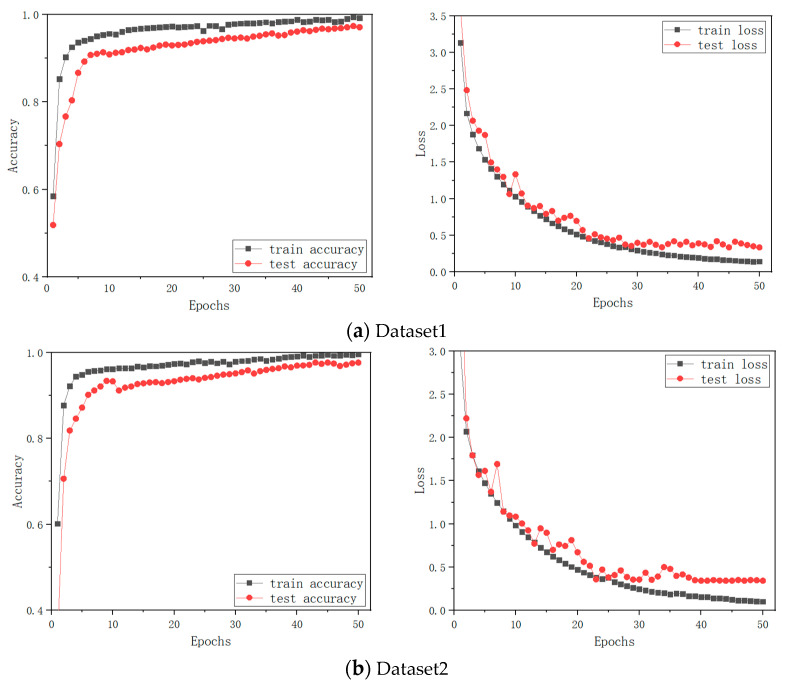

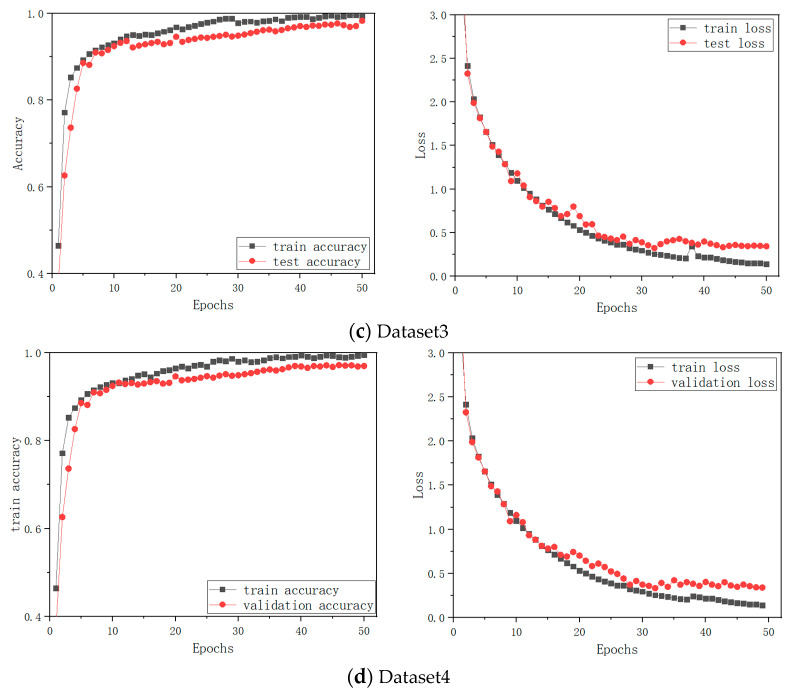
Training and testing results of MCMS-CNN.

From Figure 8, the converted RGB images of different fault conditions look completely different, which provides an intuitive way to classify them. After the RGB fault images are obtained, the proposed MCMS-CNN is applied to realize fault diagnosis. The detail of the proposed network structure is presented in Table 3. In Table 3, Conv(3 × 3 × 32) means that it is a convolution layer of 3 × 3 convolutional kernel size and 32 filters. The Maxpooling layer is adopted and its pool size is 2 × 2. The stride size of the Maxpooling layer is 2 × 2. BatchNormalization is adopted. The output size is 13, which represents the number of fault conditions. The number of training epochs is determined to be 50. The dropout rate is 0.5 to prevent overfitting. The learning rate is initialized to 0.001. The training and testing results of the four divided datasets of one trial are shown in Figure 9.

From Figure 9, it can be observed that the performance of MCMS-CNN on four datasets is satisfactory. From the results, both training and testing accuracy curves rise rapidly. The training accuracy stabilizes after 35 epochs, and the testing accuracy stabilizes after 40 epochs. The loss functions of training and testing drop rapidly in the first 20 epochs. As the number of epochs increases, the loss functions immediately stabilize only at the fiftieth epoch.

### 4.3. Comparison of the Performance of MCMS-CNN and Single-Scale-Based CNNs

**Figure 10:**
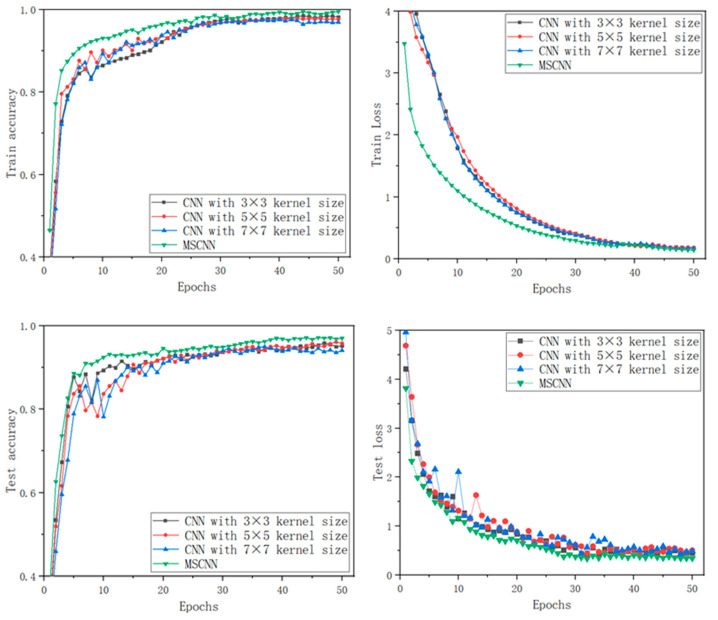
Results of three single-scale-based CNNs with different kernel sizes and MCMC-CNN.

To illustrate the efficiency of the proposed MCMS-CNN, a total of three comparison models are designed, which adopt parallel three-channel convolution operation consistent with the algorithm in this paper. The difference is that each of these models adopts convolution kernels of fixed and single size in the channels. Specifically, model 1 (CNN with 3 × 3 kernel size) adopts a 3 × 3 convolution kernel in each convolutional channel, model 2 (CNN with 5 × 5 kernel size) adopts 5 × 5 convolution kernel in each convolutional channel, model 3 (CNN with 7 × 7 kernel size) adopts 7 × 7 convolution kernel in each convolutional channel. All the CNNs are evaluated on the same dataset with 50 running epochs for one fold. Figure 10 shows the results. Table 4 elucidates their performance.

The training accuracy curve in Figure 10 depicts that the proposed MCMS-CNN achieves high training accuracy in 35 epochs. The accuracy curve rises faster than those of single-scale-based CNN models and remains stable after reaching the highest accuracy. CNN models with 5 × 5 and 7 × 7 kernel sizes have similar training accuracy curves in the 40th epochs. However, as the training accuracy curves reach a stable state, the accuracy of CNN model with 7 × 7 kernel size is slightly lower than that of CNN model with 5 × 5 kernel size. The training accuracy curve of a CNN model with 3 × 3 kernel size rises fast in the first 10 epochs, then increases slower than other models, and finally reaches the maximum training accuracy after 40 epochs. Overall, the accuracy curves on the testing set are consistent with those on the training set. MCMS-CNN still surpasses the other models with outstanding performance.

From the results in loss function, the loss function of MCMS-CNN is considerably superior to those of other CNNs on the training and testing sets. After only 30 epochs, the test loss function of MCMS-CNN drops rapidly and remains stable. In comparison, the test loss functions of the three single-scale-based CNNs decline slower, of which the slowest one is the CNN model with a 7 × 7 kernel size. In terms of the final stable state, MCMS-CNN reaches a stable state after 30 epochs, and the CNN model with 7 × 7 kernel size reaches a stable state after 45 epochs. In terms of training loss function curves, the loss function curve declines faster than those of single-scale-based CNN models. The loss functions of the remaining three models decline similarly.

The comparison results of running different models 10 times are shown in Table 4. The MCMS-CNN achieves a satisfactory performance, in which the mean classification accuracy is up to 97.2%. It is superior to all other single-scale-CNNs. The classification accuracies of the CNN models with 3 × 3, 5 × 5, and 7 × 7 kernel sizes are only 95.8%, 96.1%, and 94.6%, respectively. The results show that only single scale features are extracted from the generated RGB images, resulting in the inability to obtain rich features at different scales to adapt diverse fault types. By contrast, MCMS-CNN can extract more identifiable and robust features of diverse scales.

Therefore, MCMS-CNN has a stronger adaptive feature-extraction and classification capability on small datasets with a low resolution composed of color pixels than the single-scale strategy.

### 4.4. Influence of MCMS-CNN Models Combined with Different Channels and Scales on Classification Results

Based on the comparative experimental analysis in the previous section, it is easy to see that when the number of channels is fixed; concatenated convolution kernels of different scales produce excellent classification results. In this section, we discuss the influence of different numbers of channels for bearing-fault classification. To verify the influence of the different multiple channel, three different MCMS-CNN models, which are 2channel-2scale, 3channel-3scale, and 4channel-4scale, are proposed. All three models have a similar structure to the MCMS-CNN proposed in this article. The only differences among these models are the number of parallel convolution channels and the sizes of convolutional kernels. As such, 2channel-2scale refers to a parallel two channel CNN, in which the sizes of each channel convolution kernel are 3 and 5. Accordingly, 3channel-3scale refers to a parallel three channel CNN, in which the sizes of each channel convolution kernel are 3, 5, and 7 (the model in this paper). Furthermore, 4channel-4scale refers to a parallel four channel CNN, in which the sizes of each channel convolution kernel are 3, 5, 7, and 9.

**Figure 11:**
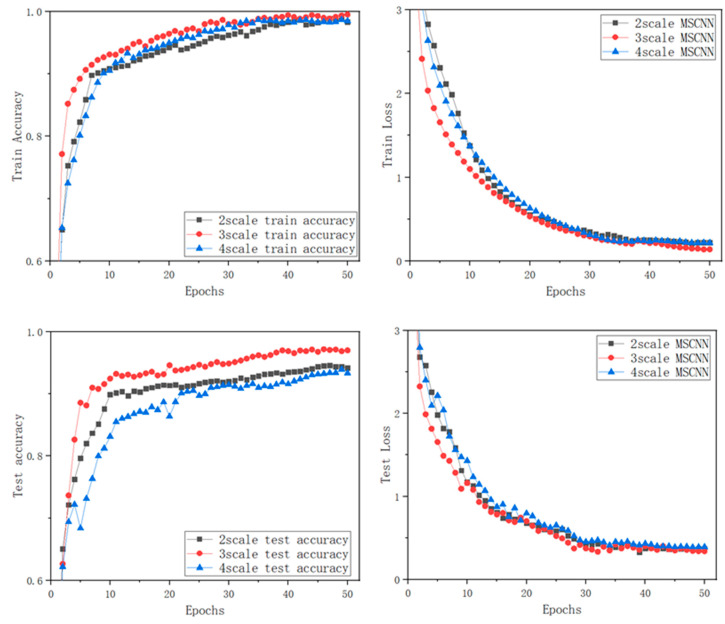
Results of different, multiple-scale MCMS-CNN.

In case of dataset4, the comparison experiments are conducted under the condition that the experimental environment, the experimental parameter settings, and the dataset all remain unchanged. To highlight the difference in the experimental results, the accuracy coordinates of the training and testing set are adjusted to an interval of 0.6–1, and the loss function curves are adjusted to an interval of 0–3, as shown in Figure 11.

The training accuracy curves demonstrate that MCMS-CNNs of different scales achieve state-of-the-art training accuracy after 50 epochs, and the overall trend is relatively similar. However, the training accuracy of 3channel-3scale CNN is slightly superior to those of the two other MCMSCNNs. From the results in training loss, the loss functions of the three models drop rapidly in the first 20 epochs. Among them, the declining speed of the 3channel-3scale CNN is slightly ahead of those of the two other MCMS-CNNs. The worst performance is shown by the 4channel-4scale CNN. The decline rate is slightly slower than those of the two other MCMS-CNNs. The testing accuracy curve of the proposed method is significantly higher than that of the other two models. From the test loss value comparison, these models gradually become smooth with the increase in iterations. The proposed model converges slightly faster, and reaches a stable convergence state at the 30th epoch.

The comparison results on the classification performance by different MCMS-CNNs are shown in Table 5, which are obtained by running the three models 10 times. Thus, 3channel-3scale CNN has the best performance and achieves the highest mean accuracy of 97.2%, about 2 percentage points higher than the 2channel-2scale CNN and more than 3 percentage points higher than the 4channel-4scale CNN. Although multiple scales can obtain complementary and abundant fault information, the classification effect of the dataset in this article does not improve with the increase in scale number. The classification performance on the 3channel-3scale CNN (the proposed method in this paper) is the best.

### 4.5. Comparison of Single-Sensor Data and Multisensor Fusion Data Using STRIM

**Figure 12:**
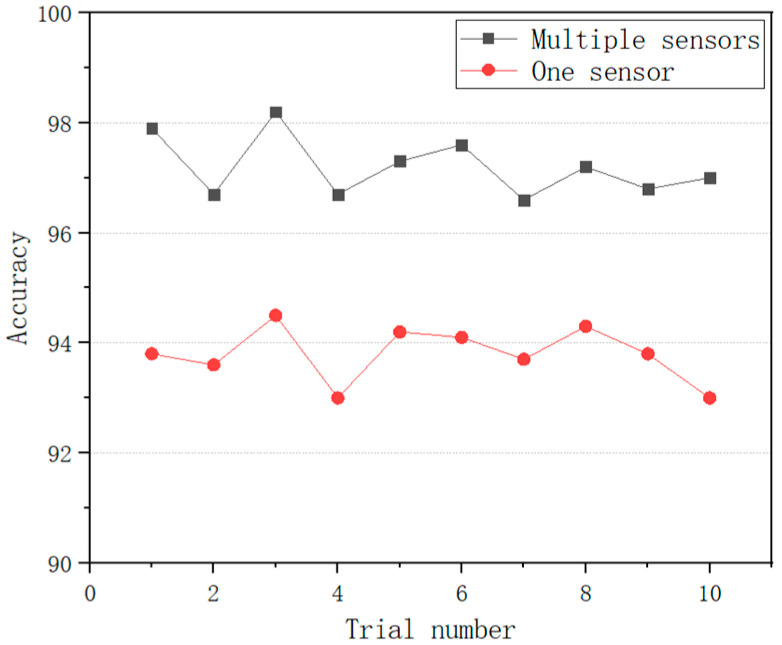
Diagnosis results of 10 trials.

To illustrate the effectiveness of the STRIM method, the signal from only one sensor (DE) is used for comparison. The time-domain signal of one sensor (DE) is transformed into gray images using STIM according to the method of [44]. Next the gray images are fed to the MCMS-CNN for fault classification. As shown in Figure 12, 10 trials are performed to diagnose the bearing conditions. The average test accuracy is 97.2% via multisensory fusion, which is higher than the average test accuracy of 93.8% using only one sensor. The result illustrates that the RGB images processed by STRIM contain all fault information, and the multi-sensor fusion method provides high-quality feature sources for subsequent feature extraction, and has a better robustness and a higher accuracy.

### 4.6. Comparison with Other Methods

Classification accuracy is regarded as an index, and MCMS-CNN is compared with several mainstream models, which are listed in Table 6. In addition to presenting the classification accuracy, the division results of the dataset used for each model and the existence of similar bias are also discussed, which is also crucial for comparison. The comparison methods are DL methods that use time–frequency domain transform to extract features. Youcef Khodja et al. [11] utilized CNN and VSI for bearing-fault classification. The classification of 10 fault conditions was realized, achieving a test accuracy of 97.27%. David Verstraete et al. [45] adopted three time-frequency analysis methods, namely, short-time Fourier transform (STFT), wavelet transform (WT), and Hilbert–Huang transform (HHT), to generate images. The images generated by these three methods were then fed to the CNN to achieve classification. In paper [46], the EDAEs was introduced to realize the fault classification of 12 failure conditions, and obtained a prediction accuracy of 97.18%.

From the comparison, the proposed method outperforms the above mainstream intelligent fault-diagnosis methods in the number of classified fault conditions. The classification accuracy is relatively superior. However, the problem of similarity bias is fully considered, which leads to overoptimistic results. If the similarity bias is ignored, the classification accuracy of the proposed method can reach 99.8%, proving the effectiveness of the proposed model based on STRIM and MCMS-CNN. Moreover, the comparison methods have achieved good results in the bearing-fault classification task, but the diagnosis effect depends on the complex signal processing process. For example, the parameter selection of a wavelet packet will directly affect the quality of extracted features, increase the uncertainty of fault analysis, and reduce the intelligence of deep learning. However, the original vibration data contain a large amount of state information related to the operation of mechanical equipment. The proposed method can directly use the original information in vibration signals, and does not require a complex signal preprocessing process, which can more intuitively and accurately reflect the status of mechanical equipment, so as to achieve end-to-end fault diagnosis.

Strictly speaking, due to the problem of dataset division and similarity bias, the above comparison experiment is unfair to some extent. In order to make the comparison more convincing, we adopt the dataset division scheme in this paper and add comparative analysis to some classical algorithms. These methods include KNN, SVM, and random forest (RF). In Table 7, detailed results of comparison are given, including the hyperparameter settings of the comparison algorithm. The parameter settings of the proposed method are given in detail in Section 4.2.

Not surprisingly, compared with the classical machine learning methods, the proposed model shows the best performance in bearing-fault classification. Although the results on KNN and SVM are not satisfactory, the better results obtained on RF show that the images obtained by STRIM provide a good basis for subsequent fault classification.

### 4.7. Discussion

The proposed method based on STRIM and MCMS-CNN is validated on the CWRU bearing dataset. The signals from multiple sensors are converted into RGB images, which integrates the period information and all sensor data associated with fault conditions. Compared with single-scale CNNs, the multichannel and multiscale strategy can extract features at different scales using parallel convolutional layers, which enables complementary and abundant features to be obtained. Compared with the STIM, the proposed STRIM retains the full information of the fault signal on which MCMS-CNN can be used to achieve perfect classification. To illustrate the effectiveness of the proposed method, we compare MCMS-CNN with other classic machine learning methods on the same dataset. Obviously, the performance of MCMS-CNN is superior to other models, exhibiting potential.

## 5. Conclusions

Since the existing signal-to-image methods cannot fusion multiple sensor data of the rolling bearings, traditional signal-processing algorithms usually rely on much diagnostic expertise and knowledge. A novel STRIM method for converting time-series data into multichannel RGB images is proposed. This method realizes the feature fusion of multiple sensors. The feature fusion is achieved at the data level and combines time, space, and period information, retaining all fault information. STRIM does not rely on complicated signal-processing algorithms and prior knowledge. The MCMS-CNN is then applied to mine the useful information hidden in the integrated RGB images. By introducing the multichannel and multiscale strategy, the capability of feature extraction of different scales is ensured, and the performance of bearing-fault diagnosis is improved. Moreover, the proposed MCMS-CNN reduces the scale of the model, avoiding overfitting phenomena due to the limited samples and deep network. SGD and dropout are applied in the training process, increasing the efficiency of fault diagnosis. Through comparative experiments among single-scale-based CNNs with different kernel sizes, the experimental results demonstrate that the MCMS-CNN can significantly outperform single-scale-based CNNs in aspects of feature learning and classification performance. Compared with other intelligent fault-diagnosis models on the same bearing dataset, this novel method achieve the diagnosis of many more fault categories and is superior to other methods. The method based on STRIM and MCMS-CNN provides an idea of the mechanical data fusion of various sensors and fault diagnosis, which is promising for handling more complex industrial systems in the future. Only three sensors’ data are discussed in this paper; data fusion and image conversion for more complex and multi-sensor data will be the direction of our subsequent research.

## Figures and Tables

**Table 1:** Fault conditions.

Label	Condition Type	Defect Severity (mm)	Sample Number
1	BD	0.18	300
2	BD	0.36	300
3	BD	0.54	300
4	IR	0.18	300
5	IR	0.36	300
6	IR	0.54	300
7	OR@3	0.18	300
8	OR@6	0.18	300
9	OR@12	0.18	300
10	OR@6	0.36	300
11	OR@3	0.54	300
12	OR@6	0.54	300
13	OR@12	0.54	300

**Table 2:** Sample division results of dataset1.

Condition Type	Total Samples	Training Samples	Testing Samples
BD-0.18	1200	900	300
BD-0.36	1200	900	300
BD-0.54	1200	900	300
IR-0.18	1200	900	300
IR-0.36	1200	900	300
IR-0.54	1200	900	300
OR@3-0.18	1200	900	300
OR@6-0.18	1200	900	300
OR@12-0.18	1200	900	300
OR@6-0.36	1200	900	300
OR@3-0.54	1200	900	300
OR@6-0.54	1200	900	300
OR@12-0.54	1200	900	300

**Table 3:** Layer configurations of MCMS-CNN.

Layers	CNN Operator	Stride
Channel 1	Conv (3 × 3 × 32)	1 × 1
	Maxpooling (2 × 2)	2 × 2
	BatchNormalization	
Channel 2	Conv (5 × 5 × 32)	1 × 1
	Maxpooling (2 × 2)	2 × 2
	BatchNormalization	
Channel 3	Conv (7 × 7 × 32)	1 × 1
	Maxpooling (2 × 2)	2 × 2
	BatchNormalization	
Output layer	Softmax	

**Table 4:** Results of different kernel sizes of CNNs and MCMS-CNN (%).

Run NO.	CNN with 3 × 3 Kernel Size	CNN with 5 × 5 Kernel Size	CNN with 7 × 7 Kernel Size	MSMC-CNN
1	96.3	95.5	95.0	97.9
2	96.4	96.0	94.5	96.7
3	96.4	96.4	94.8	98.2
4	95.1	96.8	95.2	96.7
5	95.8	96.2	94.0	97.3
6	96.0	95.6	94.2	97.6
7	95.4	96.1	94.1	96.6
8	96.1	95.7	93.9	97.2
9	95.3	96.3	95.2	96.8
10	95.2	96.4	95.1	97.0
Mean	95.8	96.1	94.6	97.2

**Table 5:** Comparison results of different channels and scales MCMS-CNN (%).

Test Accuracy	2scale MCMS-CNN	3scale MCMS-CNN	4scale MCMS-CNN
Max	95.1	98.2	94.5
Min	94.2	96.6	93.3
Mean	94.6	97.2	93.8

**Table 6:** Comparison results of different methods (%).

Method	Number of Fault Conditions	Training Samples	Testing Samples	Similarity Bias	Classification Accuracy
CNN + VSI	10	1580	780	yes	97.27
CNN + STFT	4	8827	3783	yes	98.10
CNN + WT	4	8827	3783	yes	98.80
CNN + HHT	4	8827	3783	yes	86.50
EDAEs	12	2400	1200	yes	97.18
Proposed method	13	11,700	3900	no	97.20

**Table 7:** Comparison results of different methods using the same division strategy (%).

Method	Hyperparameters	Values	Classification Accuracy
KNN	Number of neighbors	15	67.4
SVM	RBF Kernel γ $\log_{10}\left(\cdot \right)$	−3	72.8
RF	Number of trees (estimators)	100	
	Number of features for split	5	91.1
